# Scrotal extratesticular schwannoma: a case report and review of the literature

**DOI:** 10.1186/1471-2490-14-32

**Published:** 2014-04-28

**Authors:** Giovanni Palleschi, Antonio Carbone, Jessica Cacciotti, Giorgia Manfredonia, Natale Porta, Andrea Fuschi, Cosimo de Nunzio, Vincenzo Petrozza, Antonio Luigi Pastore

**Affiliations:** 1 Sapienza University of Rome, Department of Medical and Surgical Biotechnologies, Unit of Urology, ICOT, Via Franco Faggiana 1668, 04100 Latina, Italy; 2 Uroresearch Association, non profit association, Via Franco Faggiana 1668, 04100 Latina, Italy; 3 Department of Medical and Surgical Biotechnologies, Histopathology Unit, ICOT Latina, via Faggiana 1668, Latina, Italy; 4 ICOT Hospital, CADI Centre, via Faggiana 1668, Latina, Italy; 5 Sapienza University of Rome, Department of Urology, Sant’Andrea Hospital, Rome, Italy

**Keywords:** Scrotal schwannoma, Diagnosis, Histopathology

## Abstract

**Background:**

Schwannomas are tumours arising from Schwann cells, which sheath the peripheral nerves. Here, we report a rare case of left intrascrotal, extratesticular schwannoma. Although rare, scrotal localisation of schwannomas has been reported in male children, adult men, and elderly men. They are usually asymptomatic and are characterised by slow growth. Patients generally present with an intrascrotal mass that is not associated with pain or other clinical signs, and such cases are self-reported by most patients. Imaging modalities (such as ultrasonography, computed tomography, and magnetic resonance imaging) can be used to determine tumour size, exact localisation, and extension. However, the imaging findings of schwannoma are non-specific. Therefore, only complete surgical excision can result in diagnosis, based on histological and immunohistochemical analyses. If the tumour is not entirely removed, recurrences may develop, and, although malignant change is rare, this may occur, especially in patients with a long history of an untreated lesion. Thus, follow up examinations with clinical and imaging studies are recommended for scrotal schwannomas.

**Case presentation:**

A 52-year-old man presented with a 3-year history of asymptomatic scrotal swelling. Physical examination revealed a palpable, painless, soft mass in the left hemiscrotum. After surgical removal of the mass, its histological features indicated schwannoma.

**Conclusions:**

Schwannoma should be considered in cases of masses that are intrascrotal but extratesticular. Ultrasonography provides the best method of confirming the paratesticular localisation of the tumour, before surgical removal allows histopathological investigation and definitive diagnosis. Surgery is the standard therapeutic approach. To prevent recurrence, particular care should be taken to ensure complete excision. This case report includes a review of the literature on scrotal schwannomas.

## Background

Schwannomas are tumours arising from Schwann cells, which sheath the peripheral nerves. Schwannomas can develop in any region of the human body, either sporadically or in association with neurofibromatosis [1]. Most clinical findings associated with schwannoma are not specific to the disease, because schwannomas grow slowly and do not result in symptoms until they enlarge and compress the surrounding structures [2]. The exact incidence of schwannoma is not known [3]. Scrotal localisation of schwannoma is rare, but has been the occasional subject of reports in the literature [4,5]. We recently treated a case of intrascrotal, extratesticular schwannoma, which was found in an adult man who had a 3-year history of an asymptomatic lesion in the left hemiscrotum. Here, we describe the case with a complete report, including intraoperative findings. We also provide a review of the literature, which was performed to collect and summarise the present state of knowledge on this type of tumour.

## Case presentation

### Clinical history

A 52-year-old man presented with a 3-year history of asymptomatic scrotal swelling. He had a body mass index of 28 kg/m2 at the time of presentation. The patient reported that his scrotum had significantly enlarged during the last 6 months. Physical examination revealed a palpable, painless, soft mass in the left hemiscrotum, approximately the size of an egg. Further, an adhesion had formed between the mass and the left testicle. The lesion appeared not to be attached to the skin of the scrotum or the underlying tissues. The abdominal examination findings were unremarkable, and rectal exploration showed enlargement of the prostate gland without any palpatory pathological findings. The patient did not report any systemic symptoms. Accordingly, the initial diagnostic hypothesis was suspicion of a left testicular tumour. Written informed consent was obtained from the patient for publication of this case report and any accompanying images.

### Imaging

**Figure 1:**
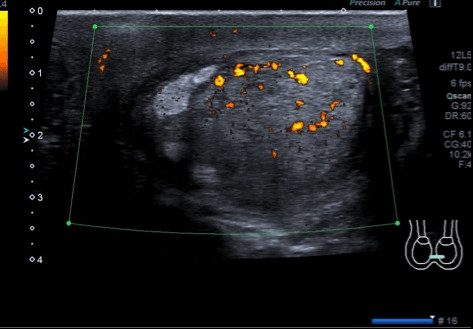
**Ultrasonography and colour Doppler examination of the schwannoma.** The lesion appeared inhomogeneous, was partially hypoechogenic, and had poor hypervascularisation.

**Figure 2:**
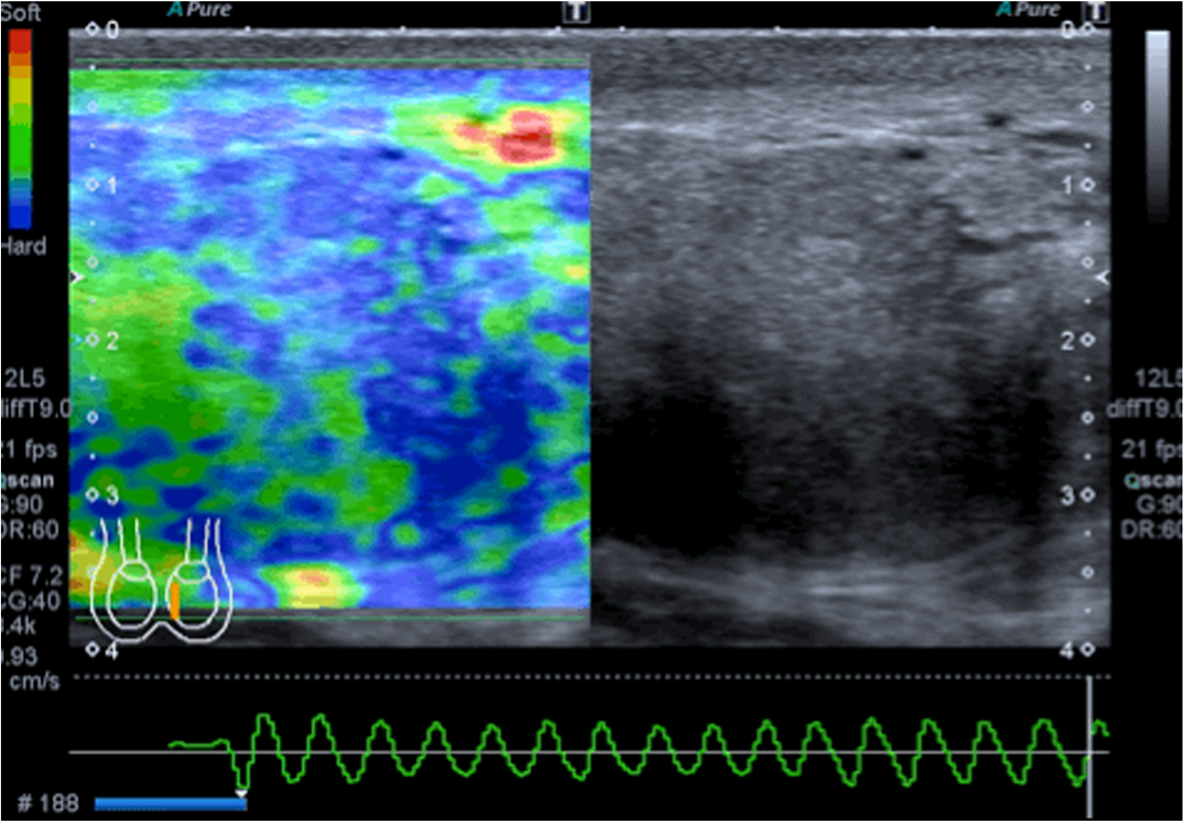
**Elastographic investigation of the lesion.** Quantitative elastography showed that the lesion had a low elastic modulus, as represented by the green area. After compression induced by the operator, there was no modification in the elastographic waves.

The patient underwent scrotal ultrasonography (US), which revealed a hypoechoic solid mass. The mass was 4 × 7 cm in size and had a nodular appearance. It was found in the mid-left hemiscrotum, behind the left testicle, which appeared reduced in size (Figure 1). Left varicocele was observed, whereas hydrocele was absent bilaterally. Colour Doppler evaluation showed poor perilesional and intralesional hypervascularisation. Quantitative elastography revealed that the lesion had a low elastic modulus relative to the left testicular parenchyma and low intralesional vascularity (Figure 2). The patient’s right testicle was normal. To better examine the patient, contrast-enhanced computed tomography (CT) of the abdomen, scrotum, and chest was performed. The scan identified a 7.1 × 3.9 cm mass, which was localised medially in the left testicle, without extension into the tunica vaginalis. Lymphadenopathy was not present.

### Laboratory findings

Blood tests showed normal levels of alpha-fetoprotein, beta-human chorionic gonadotropin, and carcinoembryonic antigen. Prostate specific antigen levels were also normal.

### Therapeutic approach

**Figure 3:**
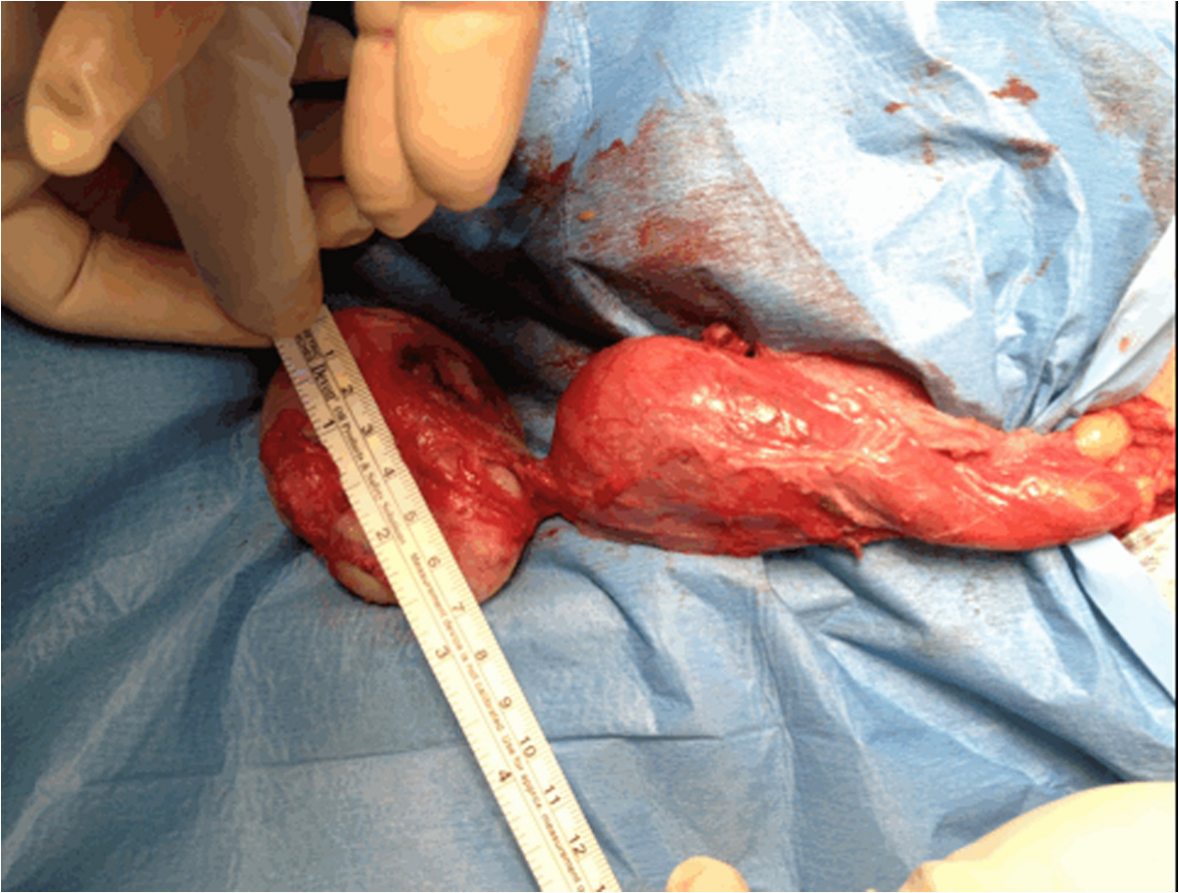
**Intraoperative appearance of the lesion.** The mass presented with some adhesions to the left testicle, but did not infiltrate the testicle.

Based on this evidence, we decided to surgically remove the mass, and the patient agreed. A left inguinal 4-cm incision was made, the spermatic cord was clamped, and the testicle was tractioned from the left inguinal ring. The mass presented with some adhesions to the left testicle, but did not infiltrate the testicle (Figure 3). Surgical removal appeared to be simple and was performed using monopolar scissors in a few minutes. The spermatic cord was then released, and the left testicle was pushed back into the scrotum. The patient was discharged 48 hours after surgery, without complications. After 12 months of follow-up, physical examination and scrotal ultrasound were negative.

### Macroscopic findings

**Figure 4:**
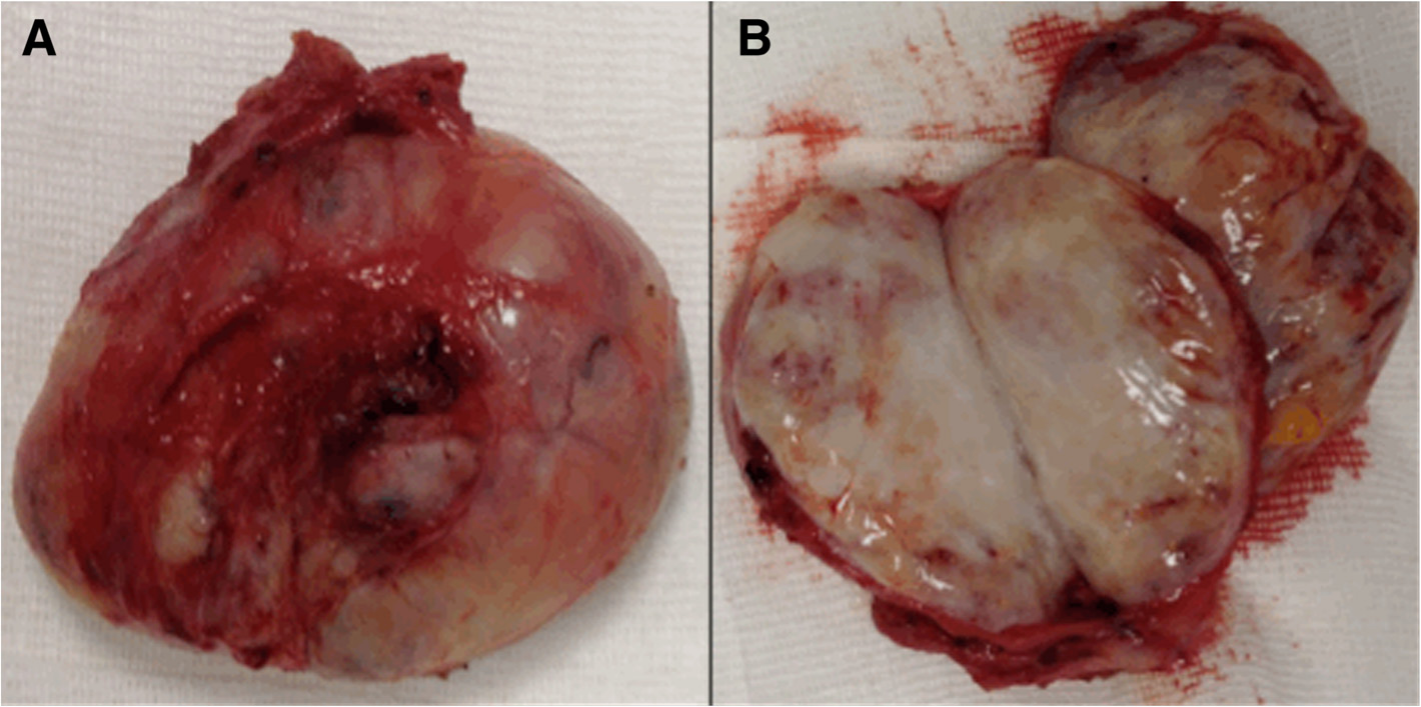
**Macroscopic examination and sectioning of the lesion. (A)** Macroscopic examination after surgical removal showed a white mass that was soft and had a regular, translucent external surface. **(B)** After sectioning, the specimen was multinodular in appearance, with haemorrhagic areas.

The surgical specimen was immediately measured and found to be 7.1 × 3.8 cm. It comprised a white mass, which was soft and had a regular and translucent external surface (Figure 4A). The specimen was sectioned at the midline along the longest diameter. The inner tissue presented a multinodular appearance with haemorrhagic areas (Figure 4B).

### Microscopic findings

**Figure 5:**
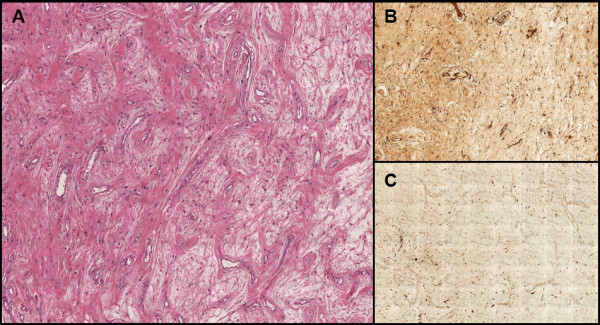
**Microscopic findings. (A)** Proliferation was characterised by spindle elements with elongated hyperchromatic nuclei and poorly eosinophilic cytoplasm, separated by abundant oedematous fluid. These elements were occasionally arranged concentrically around vessels with thin walls (magnification × 4). **(B)** The cells show intense immunoreactivity for vimentin (magnification × 20) and **(C)** S-100 protein (magnification × 20).

Considerable cellular proliferation was observed, comprising spindle elements with elongated hyperchromatic nuclei and poorly eosinophilic cytoplasm, separated by abundant oedematous fluid. A myxoid appearance was noted focally (the so- called ‘Antoni B pattern’). These elements were occasionally arranged concentrically around vessels with thin walls. Areas of necrosis and mitoses were not observed (Figure 5A). Immunohistochemically, the tumour cells showed intense immunoreactivity for S-100 protein and vimentin. Further, tumour cells were negative for smooth-muscle actin, desmin, and CD-34, supporting the neural differentiation of the tissue (Figure 5B and C). These histological features were suggestive of schwannoma.

### Literature review and comparison with previous cases (Table 1)

**Table 1:** Literature review with onset symptoms, management, and final diagnosis

**Authors**	**Onset symptoms**	**Management**	**Diagnosis**
Sighinolfi MC et al. [2]	Presence of a small and painless swelling with elastic consistency	Orchifunicolectomy	Intratesticular neurilemoma
Chan PT et al. [3]	Asymptomatic scrotal swelling	Surgical excision	Extratesticular schwannoma
Arciola AJ et al. [4]	Supratesticular intrascrotal mass clinically mimicking a spermatocele	Surgical excision	Intrascrotal schwannoma
Fernandez MJ et al. [5]	Intrascrotal giant painless mass	Surgical excision	Giant neurilemoma of the scrotum
Kim YJ et al. [6]	Episode of multiple slowly growing masses in the scrotum	Surgical excision	Schwannomas of the scrotum
Matsui F et al. [9]	Painless, solid and elastic-hard scrotal mass	Tumor resection	Giant scrotal schwannoma
Safak M et al. [10]	Painless, solid scrotal mass	Surgical excision	Intrascrotal extratesticular malignant schwannoma
Muzac A and Mendoza E [11]	Inguinal scrotal painless solid mass	Surgical excision	Malignant schwannoma

Scrotal masses require a precise diagnosis to prevent therapeutic errors. Among various types of intrascrotal extratesticular lesions, schwannomas have been described previously and have always represented a diagnostic challenge for clinicians. During differential diagnosis of these tumours, clinicians must also consider benign and malignant neoplasms of supporting structures of the scrotum: leiomyoma, leiomyosarcoma, and adenosarcoma [3].

The clinical history of the disease was similar in each of the previous reports that we examined [3-6]. Each of the subjects presented with painless scrotal swelling, which had been present for between a few months [3] and 3 years, as in our case. The ages of patients varied considerably across the reports, although older ages (>60 years) were most common. Physical examination usually revealed a soft mass without adhesion to the scrotal skin or the surrounding tissues. Yet, examining the patient does not entirely clarify whether the lesion originated from the testicle or another location. In previously reported cases, therefore, US, magnetic resonance imaging (MRI), and CT have variously been used to arrive at more exact diagnoses [2,3,6]. However, none of these options have allowed schwannoma to be identified, because there is no pathognomonic finding for this disease [6]. On US, schwannoma generally appears as a well-circumscribed mass with a hypoechoic pattern [2], and on colour Doppler imaging, poor hypervascularisation is noted. In our case, we also performed quantitative elastography, which showed that the mass had a lower elastic modulus than the testicle, as the result of a higher tissue density, which is typical of tumours. However, despite some evidence that it contributes to a diagnostic algorithm for scrotal masses, the value of elastography is still under investigation and should only be used if combined with standard methods [7,8]. Nevertheless, because of the lack of specific findings that can identify schwannomas, many clinicians have used MRI and CT to confirm the extratesticular localisation of the lesion. For this purpose, MRI appears to be more capable of identifying the tumour and distinguishing it from testicle parenchyma [3], showing a peripheral rim-enhancement of the pathologic tissue in T2 weighted sequences [1]. In addition, laboratory examinations are not helpful for diagnosing scrotal schwannoma, and none of the previous reports found alterations to testicular tumour markers. Surgical excision remains the mainstay of treatment for scrotal schwannomas, as the authors of all previous reports have noted. In a previous case, the schwannoma was localised intratesticularly and required orchiectomy [2]. In another case, scrotectomy was required due to an extension of the tumour superficial to the tunica vaginalis, testis, and corpus spongiosum [3]. In all cases, definitive diagnosis was achieved only by histopathological examination, combined with positive immunostaining for S-100 protein and negative staining for CD-34 [2,3]. Immunohistochemical evidence is needed for the diagnosis of schwannoma. However, fine-needle aspiration biopsy cannot be considered helpful, even though it has been performed in some previously reported cases; information on the tissue’s architecture is required for diagnosis, but cannot be obtained from the cytology specimen [3]. Macroscopically, the largest scrotal schwannoma reported in the literature was described by Matsui et al., who surgically removed a 13 × 7.5 × 3.0 cm intrascrotal tumour, which weighed 285 g [9]. Cases of multiple scrotal schwannomas have also been reported. Kim et al. reported multiple schwannomas of the scrotum (with the largest lesion measuring 3.5 cm) in a 67-year-old man. In this case, some lesions had invaded the penile root without reducing erectile function or causing penile deviation. The author reported no recurrence for at least 6 months after surgery. For patients with the systemic disease of schwannomatosis, the development of a single or multiple scrotal masses should lead the clinician to suspect peripheral localisation of schwannoma. In another case, reported by Ikary et al., a scrotal schwannoma developed in a 66-year-old man who had previously undergone surgical excision of a brain tumour, which itself originated from the glossopharyngeal nerve [1]. The patient’s histological diagnosis was schwannoma. Therefore, regular surveillance is needed for patients with schwannomatosis, because they are at a high risk of developing multiple schwannomas. However, follow-up is also required for patients who have undergone surgical removal of a scrotal schwannoma. Indeed, as has generally been reported for schwannomas located in other regions of the body, incomplete removal can be followed by local recurrence, even many years later. Safak et al. reported a case of late recurrence in the scrotum in 1998. The authors reported late local recurrence after treatment of an intrascrotal extratesticular malignant schwannoma with rhabdomyoblastic features in an adult man [10]. Muzac and Mendoza reported a case of malignant schwannoma in 1992 [11]. Malignant degeneration of schwannomas is extremely rare, and when present, such cases have a sarcomatoid-like behaviour. The diagnosis of malignant peripheral nerve sheath tumour lacks standardised criteria and is usually based on evaluations of mitosis, pleomorphism, and blood vessel infiltration. Therefore, it is very important to remove the tumour completely, which allows the most precise histopathological investigation. From a histopathological perspective, the microscopic appearance of schwannoma is distinctive, with 2 recognisable patterns, which are not always present together: Antoni A areas and Antoni B areas. Antoni A areas are composed of compacted spindle cells that are arranged in palisades or in an organoid arrangement (Verocay bodies). Antoni B areas consist of tumour cells suspended in a myxomatous matrix that may appear microcystic. Several histological variants have been described previously: cellular, glandular, epithelioid, and ancient, which involves bizarre hyperchromatic nuclei without mitosis [3]. However, each of these variants is benign, and the ancient type is rare, especially in the scrotum (most cases occur in the head and neck region).

## Conclusion

Our experience with the reported case and our review of the literature strongly suggest that schwannoma should be considered in cases involving masses that are intrascrotal and extratesticular. US (followed by MRI) is the best modality with which to confirm the paratesticular localisation of the tumour, before surgical removal allows histopathological investigation and definitive diagnosis. Surgery is the standard therapeutic approach, which allows the testicle to be spared in most cases. To prevent recurrence, particular care should be taken to ensure complete excision, especially in cases that involve large or multiple masses. Eventhough it is hard to establish a specific follow-up plan considering the limited number of cases reported in literature, basing on all the experiences reported in literature, in case of benign lesions completely removed it might be suggested a clinical post-operative evaluation at 6 and 12 months. Differently, in case of histopathological suspicious or clear evidence of malignancy, a MRI after 6 months from surgery should be proposed.

Early diagnosis can help limit the extent of surgical excision that is required. Further, early diagnosis can prevent the onset of malignant degeneration, which is rare, but has been reported in the literature.

### Consent

Patient has given his consent for the case report to be published. A copy of the written consent is available, at anytime, for review by the Editor of this journal.

## Abbreviations

CT: Computed tomography; MRI: Magnetic resonance imaging; US: Ultrasound.

## Competing interests

No financial support has been received for this study, and none of the authors have any conflicts of interest pertinent to the content of the manuscript.

## Authors’ contributions

AC and VP ideated the study. GP, NP, JC, GM, ALP acquired the data. GP and NP drafted the manuscript. AC, GP, ALP, VP critically revised the manuscript. All authors read and approved the final manuscript.

## Pre-publication history

The pre-publication history for this paper can be accessed here:

http://www.biomedcentral.com/1471-2490/14/32/prepub
